# Anhedonia in Semantic Dementia—Exploring Right Hemispheric Contributions to the Loss of Pleasure

**DOI:** 10.3390/brainsci11080998

**Published:** 2021-07-28

**Authors:** Siobhán R. Shaw, Hashim El-Omar, Siddharth Ramanan, Olivier Piguet, Rebekah M. Ahmed, Alexis E. Whitton, Muireann Irish

**Affiliations:** 1 Brain and Mind Centre, The University of Sydney, 94 Mallett Street, Sydney, NSW 2050, Australia; Siobhan.Shaw@sydney.edu.au (S.R.S.); hashim.el-omar@sydney.edu.au (H.E.-O.); siddharth.Ramanan@mrc-cbu.cam.ac.uk (S.R.); olivier.piguet@sydney.edu.au (O.P.); rebekah.ahmed@sydney.edu.au (R.M.A.); 2 School of Psychology, The University of Sydney, Sydney, NSW 2050, Australia; 3 MRC Cognition and Brain Sciences Unit, The University of Cambridge, 15 Chaucer Rd., Cambridge CB2 7EF, UK; 4 Memory and Cognition Clinic, Department of Clinical Neurosciences, Royal Prince Alfred Hospital, Sydney, NSW 2050, Australia; 5 School of Medical Sciences, The University of Sydney, Sydney, NSW 2006, Australia; 6 Black Dog Institute, University of New South Wales, Sydney, NSW 2031, Australia; a.whitton@blackdog.org.au

**Keywords:** motivation, depression, frontotemporal dementia, Alzheimer’s disease, neuroimaging, striatum

## Abstract

Semantic dementia (SD) is a younger-onset neurodegenerative disease characterised by progressive deterioration of the semantic knowledge base in the context of predominantly left-lateralised anterior temporal lobe (ATL) atrophy. Mounting evidence indicates the emergence of florid socioemotional changes in SD as atrophy encroaches into right temporal regions. How lateralisation of temporal lobe pathology impacts the hedonic experience in SD remains largely unknown yet has important implications for understanding socioemotional and functional impairments in this syndrome. Here, we explored how lateralisation of temporal lobe atrophy impacts anhedonia severity on the Snaith–Hamilton Pleasure Scale in 28 SD patients presenting with variable right- (SD-R) and left-predominant (SD-L) profiles of temporal lobe atrophy compared to that of 30 participants with Alzheimer’s disease and 30 healthy older Control participants. Relative to Controls, SD-R but not SD-L or Alzheimer’s patients showed clinically significant anhedonia, representing a clear departure from premorbid levels. Overall, anhedonia was more strongly associated with functional impairment on the Frontotemporal Dementia Functional Rating Scale and motivational changes on the Cambridge Behavioural Inventory in SD than in Alzheimer’s disease patients. Voxel-based morphometry analyses revealed that anhedonia severity correlated with reduced grey matter intensity in a restricted set of regions centred on right orbitofrontal and temporopolar cortices, bilateral posterior temporal cortices, as well as the anterior cingulate gyrus and parahippocampal gyrus, bilaterally. Finally, regression and mediation analysis indicated a unique role for right temporal lobe structures in modulating anhedonia in SD. Our findings suggest that degeneration of predominantly right-hemisphere structures deleteriously impacts the capacity to experience pleasure in SD. These findings offer important insights into hemispheric lateralisation of motivational disturbances in dementia and suggest that anhedonia may emerge at different timescales in the SD disease trajectory depending on the integrity of the right hemisphere.

## 1. Introduction

The capacity to anticipate and derive pleasure from rewarding experiences is a fundamental determinant of goal-directed behaviour in humans. A robust literature consistently implicates two major neurochemical pathways in modulating the hedonic experience, namely the mesolimbic and mesocortical dopamine pathways [1]. The mesolimbic pathway projects from the ventral tegmental area (VTA) to the ventral striatum and on to the amygdala and hippocampus [2] and is primarily related to reward motivation, reinforcement, and associative learning [3]. In contrast, the mesocortical pathway comprises connections from the VTA to the dorsal medial prefrontal cortex [4] and has been implicated in generating motivational and emotional responses to rewarding stimuli [3,5]. Importantly, both pathways converge on “hedonic hotspots” either in or closely linked to, the ventral striatum and the prefrontal cortex [6,7]. Together, these pathways support the capacity for reward-seeking as well as maintaining equilibrium between positive and negative affective states [8].

While motivational disturbances are well-established in neuropsychiatric populations, it is only relatively recently that reward processing has emerged as a topic of interest in dementia [9]. Neurodegenerative disorders offer compelling insights into the neurocognitive architecture of the brain in the context of distinct profiles of brain network dysregulation that unfold in a predictable and coordinated fashion [10]. Frontotemporal dementia (FTD) has proven particularly informative in this regard due to the canonical degradation of brain circuits specialised for the apprehension of and responsivity to salient social and emotional stimuli [11,12]. Prototypical features of the behavioural variant of FTD (bvFTD) span the full gamut of human interpersonal functioning [13] encompassing deficits in facial emotion recognition [14], dampened physiological responses to social emotions [15], decreased empathy [16], impaired detection of social faux pas [17], and compromised capacity for theory of mind [18,19]. Importantly, these impairments have been demonstrated to relate to atrophy of predominantly right-hemisphere structures, including the amygdala, caudate, anterior temporal cortex, and orbitofrontal cortex [14,16,19].

Studies exploring reward-seeking behaviours in bvFTD suggest a link between excessive reward pursuit and degeneration of brain structures in the right hemisphere. For example, Perry and colleagues reported significant associations between primary reward-seeking behaviours (e.g., food, sex) and atrophy of the right ventral putamen extending into the right pallidum in bvFTD [20]. The authors interpreted these findings in relation to emotion lateralisation models in which inhibition and withdrawal-related behaviours are ascribed to the right hemisphere and where atrophy to this hemisphere may result in a failure to inhibit reward pursuit [20]. An alternate proposal is that the excessive reward-seeking behaviours emerging in the context of right-hemisphere atrophy described by Perry and colleagues stem from a blunted sensitivity to rewarding stimuli [21]. A recent study demonstrated a marked attenuation in the capacity to experience pleasure in FTD, attributable to dysregulation of an extended frontostriatal network typically implicated in reward processing [22]. Importantly, the neuroanatomical signature of anhedonia in FTD was found to be predominantly right-lateralised converging on orbitofrontal, paracingulate, and insular cortices, as well as the right putamen [22]. These insights resonate with reports of right-sided frontoinsular involvement in canonical features of reward insensitivity in bvFTD, such as overeating and sweet food cravings [23,24,25,26]. As such, while debate exists regarding the extent to which reward-related behaviours are lateralised in general [27,28,29], atrophy of right-sided frontostriatal brain regions appears to be disproportionately implicated in the origin of reward-processing disturbances in FTD [30].

A population of immense interest in this regard is semantic dementia (SD), a younger-onset neurodegenerative disorder characterised by lateralised profiles of anterior temporal lobe (ATL) brain atrophy, particularly in the early stages of the disease [31]. The most common left-predominant presentation of SD displays a canonical profile of left-sided atrophy of the anterior and medial portions of the temporal lobe [32]. With disease progression, however, atrophy encroaches into the contralateral hemisphere leading to marked right anterior temporal lobe (ATL) insult [33]. The progressive degeneration of right temporal regions is now understood to herald a variety of striking behavioural and socioemotional changes in SD including loss of empathy [34,35], impaired theory of mind [36], socioemotional dysregulation [35], and behavioural rigidity [37,38]. Abnormal hedonic processing of sounds has been documented in a mixed sample of FTLD, including SD patients, correlating with grey matter atrophy in a right-lateralised network including the anteromedial temporal lobe, insula, anterior cingulate, and nucleus accumbens [39]. These regions map remarkably well onto those reported in a recent study of anhedonia in an independent mixed FTLD sample, comprising SD patients [22], suggesting a modulating role for right-sided brain regions in the genesis of anhedonia in SD.

Approximately 30% of SD cases display a right-predominant temporal lobe presentation in which the canonical presentation is one of face processing impairments (prosopagnosia) and stark socioemotional dysregulation [35,38]. Studies separating out right- versus left-predominant presentations of SD are rare yet offer immense potential to clarify the contribution of right hemispheric structures to hedonic processes. The objective of this study was to determine whether anhedonia severity is modulated by the magnitude of right-sided neural degeneration by contrasting well-characterised right- versus left-predominant cases of SD on a validated measure of anhedonia. In line with prominent theories regarding the role of the right hemisphere in depression and hedonic tone [40], we hypothesised that anhedonia severity would scale with the magnitude of right-sided atrophy in this syndrome.

## 2. Materials and Methods

A total of 88 participants were recruited for this study through FRONTIER, the frontotemporal dementia research clinic based at the Brain and Mind Centre at The University of Sydney, Australia. Twenty-eight SD patients meeting diagnostic criteria for the semantic variant of primary progressive aphasia [41] were included. Within this group, twenty patients displayed predominantly left-predominant ATL atrophy (SD-L), while eight patients displayed predominantly right-predominant ATL atrophy (SD-R), as evidenced on structural magnetic resonance imaging (MRI). We further included thirty Alzheimer’s disease patients [42] as a disease control group. Diagnoses were established by consensus among a multidisciplinary team including a senior neurologist (R.M.A.), neuropsychologist, and occupational therapist based on comprehensive clinical investigation, neuropsychological assessments, along with structural MRI.

In addition, 30 healthy older Controls were recruited from the FRONTIER volunteer database and local community groups. All Controls scored 88 or above on a global cognitive screening test, Addenbrooke’s Cognitive Examination third edition (ACE-III) [43,44], zero on the Clinical Dementia Rating Scale [45], and performed within normal limits on all behavioural and cognitive measures.

Exclusion criteria for participants included a prior history of mental illness, alcohol or other drug abuse, significant head injury, or limited English language proficiency. Patients scoring < 40 on the ACE-III (max score = 100) were excluded due to the severity of their cognitive impairment.

### 2.1. Ethics Statement

Ethics approval for this study was provided by the University of New South Wales Ethics Committee and The South Eastern Sydney Local Health District (Approval: HREC 10-126 and HREC 13-177). All participants, or their person responsible, provided informed consent in accordance with the Declaration of Helsinki.

### 2.2. Clinical and Cognitive Assessment

The ACE-III was used to determine participants’ overall level of cognitive dysfunction across subscales of Attention and Orientation, Memory, Verbal Fluency, Language, and Visuospatial abilities [43]. Behavioural changes were assessed using the Cambridge Behavioural Inventory—Revised (CBI-R) [46]. Disease duration was determined as the number of years elapsed from reported onset of first symptoms to date of testing. Disease severity was calculated using the Frontotemporal Dementia Functional Rating Scale (FRS), which provides an index of functional impairment and disease staging [47].

### 2.3. Assessment of Anhedonia

The Snaith–Hamilton Pleasure Scale (SHAPS) [48] is a widely used and validated questionnaire that assesses an individual’s capacity to experience pleasure. The questionnaire comprises 14 statements such as “I would enjoy being with my family or close friends” and “I would be able to enjoy my favourite meal”, ranked on a 4-point Likert scale (1 = Strongly Disagree; 4 = Strongly Agree). Scores range from 14 to 56, with lower scores indicating a lower level of hedonic tone, i.e., a higher level of anhedonia. Given the well-documented lack of insight in many dementia disorders [49], we modified the SHAPS to probe carer ratings of anhedonia in the patient across two time points: (i) before symptom onset and (ii) current time [22]. These two measures formed the main scores of interest for subsequent analyses.

### 2.4. Assessment of Related Mood and Motivational Disturbances

Given the multifaceted nature of anhedonia and its potential co-morbidity with other neuropsychiatric symptoms, we included independent assessments of mood and motivational disturbances. The depression subscale of the Depression, Anxiety, and Stress Scale (DASS) [50] was used to assess current levels of depression and was completed by the patient. The Motivation subscale of the CBI-R [46] was included as a validated carer-rated index of apathy.

### 2.5. Statistical Analysis

Statistical analyses for cognitive and clinical data were performed using IBM SPSS Statistics, version 27.0. Prior to undertaking any analyses, the normality of distributions was checked using Shapiro–Wilk tests and boxplots. Where variables were normally distributed, separate univariate analyses of variance (ANOVAs) were used to examine group differences on continuous demographic variables (e.g., age, education) with Sidak post hoc tests conducted to explore main effects of Group (Controls, SD-L, SD-R, Alzheimer’s disease). Chi-square tests were used to investigate group differences on categorical variables (e.g., sex).

For the carer-rated SHAPS questionnaire, a mixed model ANCOVA with total ACE-III score included as a covariate was conducted exploring main effects of Group (SD-L, SD-R, Alzheimer’s disease) and Time (Before, Current), as well as relevant interactions, using Sidak post hoc tests. Partial eta-squared values accompany all ANOVAs and ANCOVAs as measures of corresponding effect sizes. Hedge’s g is included as an index of effect size for all post hoc between-group comparisons. Finally, Pearson’s *r* correlations were used to examine associations between carer-rated SHAPS scores and relevant psychosocial (e.g., DASS-D), clinical (e.g., disease duration), and cognitive measures (e.g., ACE-III Total) in patient groups. For this purpose, we combined both SD-L and SD-R into an SD Combined group to increase the power of the analyses.

### 2.6. Image Acquisition

Seventy-three participants (15 SD-L, 7 SD-R, 23 Alzheimer’s disease, and 28 Controls) underwent structural whole-brain T1-weighted structural MRI on a 3T MRI scanner equipped with a standard quadrature 8-channel head coil. Images were acquired using the following sequences: coronal acquisition, imaging matrix 256 × 256 mm, 200 slices, 1 mm isotropic voxel resolution, echo time/repetition 2.6/5.8 msec, flip angle of 8°.

Of these participants, structural MRI for 34 individuals (47%) was acquired on a 3T Philips scanner at Neuroscience Research Australia (NeuRA), while the remaining scans (*n* = 39; 53%) were acquired on a 3T GE Discovery MR750 scanner at the Brain and Mind Centre, following the FRONTIER research group’s relocation to the University of Sydney in January 2017. A dummy variable was therefore included in all imaging analyses to control for possible variations across scanning sites. All images were subject to quality control by an experienced rater, who graded the scans in terms of image quality, head movement, and presence of white matter hyperintensities. Participants were excluded from the MRI arm of the study for the following reasons: (i) MRI contraindications (e.g., pacemaker, claustrophobia); (ii) excessive head motion during acquisition; and (iii) scan was acquired >6 months before or after SHAPS completion and, therefore, was not representative of current disease staging.

### 2.7. Voxel-Based Morphometry of Grey Matter Group Differences

Whole-brain voxel-based morphometry (VBM) analyses were employed to investigate voxel-by-voxel changes in grey matter intensity between groups, using FSL (FMRIB Software Library: https://fsl.fmrib.ox.ac.uk/fsl/fslwiki (accessed on 27 July 2021)). A standard pre-processing pipeline was implemented as per previous studies [22,51]. Briefly, this included brain extraction [52], tissue segmentation [53], and alignment of brain-extracted images to the Montreal Neurological Institute (MNI) standard space using a non-linear approach [54,55]. To validate the clinical diagnosis in each patient group, non-parametric independent t-tests were run exploring peak changes in grey matter intensity in patient groups relative to Controls, with age, education, and scanning site included as covariates of no interest. Clusters were extracted voxelwise corrected for family-wise error at *p* < 0.05. Anatomical locations of statistical significance were overlaid on the MNI standard brain with maximum coordinates provided in MNI stereotaxic space.

### 2.8. Individual Differences in Atrophy of Hedonic Hotspots and SD Disease Epicentres

To complement the VBM atrophy analyses, the magnitude and asymmetry of grey matter atrophy for select regions-of-interest (ROI) in the SD group were computed [56,57]. Eight key regions that (i) typically display early and significant atrophy in SD [58] and (ii) have been previously implicated in the emergence of anhedonia [59] were considered. These regions included the left and right (a) anterior temporal lobe (ATL), (b) orbitofrontal cortex, (c) insula, and (d) striatum. For each region, binarised masks were created using the Harvard–Oxford Cortical Atlas in FSLview (https://fsl.fmrib.ox.ac.uk/fsl/fslwiki/FSLview (last accessed on 26 May 2021) and mean grey matter intensities were extracted for SD and Control participants. Focusing on the SD group, mean grey matter intensities of each region were z-scored relative to the Control group mean. Next, for each region, a bilateral “magnitude of atrophy” index (i.e., sum of z-scored left and right intensity values) was computed, with lower scores indicating greater total bilateral atrophy relative to Controls. Finally, an “asymmetry of atrophy” index was created (i.e., subtracting z-scored intensity values of right from left region), on which positive scores suggested right-lateralised atrophy, negative scores suggested left-lateralised atrophy, and scores at/around zero indicated bilateral atrophy of relatively equal magnitude. These findings were subject only to visual inspection, not statistical analyses, and together, provide a clear snapshot of individual-level differences in the extent and laterality of regions implicated in both hedonic tone and the SD disease process.

### 2.9. Grey Matter Contributions to Anhedonia in Neurodegenerative Dementia Syndromes

Whole-brain VBM correlation analyses were run in the combined patient group (*n* = 45) to explore relationships between grey matter intensity and current carer-reported anhedonia severity (SHAPS Current scores). A covariate-only statistical model was run with ACE-III Total score, total years of education, and scanning site included as nuisance variables. A positive t-contrast was used to explore associations between grey matter intensity and SHAPS Current scores. Clusters were extracted using the voxelwise method and corrected using a false discovery rate of *q* = 0.05 [60]. This yielded a corrected *p*-value of <0.007 from the data. To further guard against false positives, statistical maps were thresholded using a cluster extent threshold of 50 contiguous voxels. Anatomical locations of statistical significance were overlaid on the MNI standard brain with maximum coordinates provided in MNI stereotaxic space.

### 2.10. Clarifying Unique Regional Contributors to Anhedonia in SD

Next, associations between grey matter atrophy and anhedonia severity were explored using multiple regression in the SD group combined. Forward stepwise regression analysis was conducted in SPSS, with mean grey matter intensity values of the left and right (a) ATL, (b) orbitofrontal cortex, (c) insula, and (d) striatum included as predictor variables and current SHAPS Current scores included as the dependent variable.

Finally, a mediation analysis was run using the PROCESS V3 package in SPSS to test if left ATL atrophy mediated the relationship between right ATL degeneration and anhedonia severity. The purpose of this analysis was to understand how the encroachment of atrophy into right temporal regions relates to individual differences in anhedonia severity in SD.

## 3. Results

### 3.1. Demographic, Clinical and Cognitive Variables

Demographic, clinical, and cognitive performance is presented in Table 1. Groups were comparable in terms of age [*F*(3,84) = 0.64, *p* = 0.59, η*_p_*^2^ = 0.02], sex distribution (χ^2^ = 0.79, *p* = 0.85), and years of education [*F*(3,84) = 1.78, *p* = 0.16, η*_p_*^2^ = 0.06]. Patient groups were comparable in terms of disease duration (years elapsed since onset of symptoms; *p* = 0.12) and functional impairment on the FRS [*F*(2,49) = 2.64, *p* = 0.08, η*_p_*^2^ = 0.10].

In terms of cognitive dysfunction, significant group differences were evident on the ACE-III Total [*F*(3,84) = 50.48, *p* < 0.0001, η*_p_*^2^ = 0.65], with all patient groups displaying profound cognitive impairments relative to Controls (all *p* values < 0.001). Direct comparisons between patient groups revealed no significant differences on the ACE-III Total (all *p* values > 0.72).

### 3.2. Neuropsychiatric and Behavioural Changes

Patient groups did not differ in terms of overall behavioural change [*F*(2,52) = 2.36, *p* = 0.12, η*_p_*^2^ = 0.08] or level of apathy [*F*(2,52) = 0.63, *p* = 0.53, η*_p_*^2^ = 0.02] as measured by the CBI-R. A significant group difference was found for participant self-reported depression severity on the DASS [*F*(3,76) = 5.57, *p* = 0.002]. Specifically, SD-R patients reported more severe depression (*p* = 0.005) in comparison to Controls, with no other differences between groups (all *p* values > 0.31).

### 3.3. Anhedonia Severity across Dementia Syndromes

**Figure 1:**
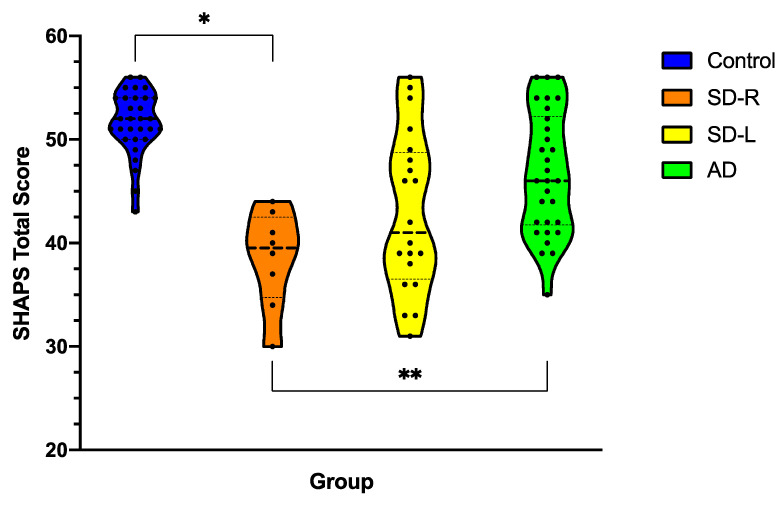
Severity of anhedonia in dementia syndromes at the current time as rated by carers on the Snaith–Hamilton Pleasure Scale (SHAPS) compared to self-rated anhedonia in Controls. Violin plots depict the distribution of data with the width of the shaded area representing the proportion of data located there. Bolded horizontal line depicts the mean score. Maximum score on SHAPS is 56, with lower scores reflecting reduced hedonic tone/greater levels of anhedonia. AD = Alzheimer’s disease (*n* = 30); SD-L = left-predominant semantic dementia (*n* = 20); SD-R = right-predominant semantic dementia (*n* = 8). Asterisks denote results that emerged as significant in the ANCOVA/MANCOVA with ACE-III included as covariate. * *p* < 0.05; ** *p* < 0.005.

Figure 1 displays current levels of anhedonia on the SHAPS for patients (carer-rated) versus Controls (self-rated). A univariate ANCOVA controlling for ACE total revealed a significant main effect of Group for current anhedonia severity [*F*(3,83) = 5.25, *p* = 0.002, η*_p_*^2^ = 0.16]. Post hoc analyses showed that, relative to Controls, SD-R patients displayed significantly higher levels of anhedonia (*p* = 0.006, mean difference = 9.69, *g* = 3.72, 95% C.I. 2.07–17.3). This was in contrast to SD-L (*p* = 0.16, mean difference = 5.3, *g* = 1.60, 95% C.I. 1.1–11.8) and Alzheimer’s disease (*p* = 0.91, mean difference = 2.0, *g* = 1.03, 95% C.I. 3.5–7.6) who were not found to differ from Controls. Post hoc direct comparison of carer SHAPS ratings across the patient groups revealed that SD-R patients showed significantly greater levels of anhedonia than Alzheimer’s disease patients (*p* = 0.004, mean difference = 7.6, *g* = 1.42, 95% C.I. 1.7–13.5) with no other differences evident (all *p* values > 0.23).

### 3.4. Changes in Anhedonia Following Dementia Onset

**Figure 2:**
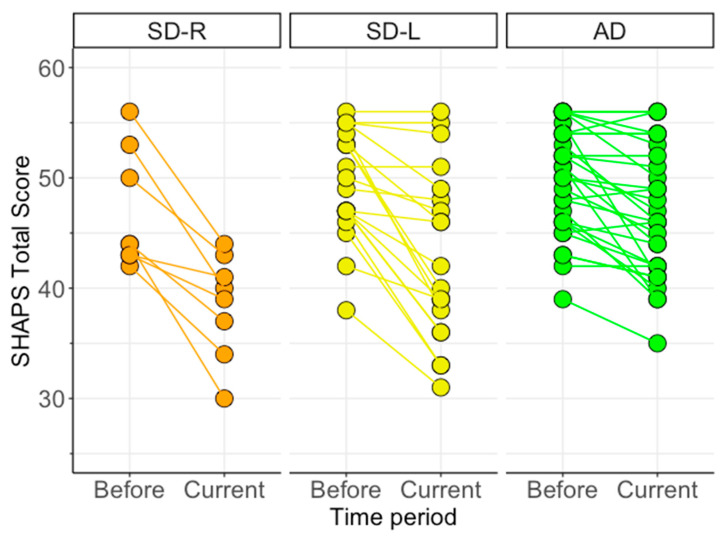
Change in anhedonia severity in dementia syndromes as rated by carers on the Snaith–Hamilton Pleasure Scale (SHAPS). Connecting lines reveal the magnitude of change between the patient’s level of anhedonia (i) pre-dementia onset (Before) and (ii) at time of assessment (Current). Maximum score on the SHAPS is 56, with lower scores reflecting greater levels of anhedonia. AD = Alzheimer’s disease (*n* = 30); SD-L = left-predominant semantic dementia (*n* = 20); SD-R = right-predominant semantic dementia (*n* = 8).

To ensure that carers’ current ratings of anhedonia were not simply capturing premorbid traits prior to dementia onset, we ran a Time (Before, Current) x Group mixed model ANCOVA on carer SHAPS scores, controlling for overall level of cognitive function on the ACE-III. Figure 2 displays carer ratings of anhedonia on the SHAPS across patient groups for Before (pre-disease onset) and Current time periods. The ANCOVA revealed a non-significant main effect of Group [*F*(2,54) = 3.09, *p* = 0.053, η*_p_*^2^ = 0.10] and a significant main effect of Time [*F*(1,54) = 5.58, *p* = 0.02, η*_p_*^2^ = 0.09]. These main effects were qualified by a significant Group x Time interaction [*F*(2,54) = 5.52, *p* = 0.007, η*_p_*^2^ = 0.17].

Post hoc tests examining the main effect of Group at each time point revealed no significant differences for premorbid SHAPS ratings (“Before”, all *p* values > 0.46) but significant group differences for current SHAPS ratings (“Current”) as outlined above (*p* = 0.002). Post hoc tests exploring the main effect of Time within each group revealed significant increases in carer-rated anhedonia from Before to Current time periods in all patient groups (all *p* values < 0.0001). Specifically, increases in carer ratings of anhedonia from premorbid to current time points were greatest in SD-R (*p* < 0.0001, mean difference = 8.3, *g* = 1.57, 95% C.I. 5.1–11.4), followed by SD-L (*p* < 0.0001, mean difference = 6.5, *g* = 1.01, 95% C.I. 4.5–8.5), and Alzheimer’s disease (*p* < 0.0001, mean difference = 3.2, *g* = 0.57, 95% C.I. 1.5–4.8) (see Figure 2).

### 3.5. Associations between Anhedonia and Clinical Measures of Interest

Table 2 displays Pearson’s *r* correlations between current carer ratings of anhedonia on the SHAPS and clinical variables of interest for the combined SD group (*n* = 28) and Alzheimer’s disease patients (*n* = 30). Briefly, carer-rated anhedonia was not significantly related to cognitive function on the ACE-III (AD: *r* = 0.11; SD: *r* = 0.33; all *p* values > 0.25) or disease duration (AD: *r* = −0.15; SD: *r* = −0.32; all *p* values > 0.08) in either patient group. In the combined SD group, anhedonia severity was associated with greater functional impairment (FRS; *r* = 0.79, *p* < 0.0001), greater behavioural change (CBI Total: *r* = −0.70, *p* < 0.0001) and higher levels of apathy (CBI-R Motivation: *r* = −0.78, *p* < 0.0001). The same pattern of associations was evident in Alzheimer’s disease albeit to a lesser extent (FRS; *r* = 0.25, *p* = 0.01; CBI-R Total; *r* = −0.51, *p* = 0.005; CBI-R Motivation; *r* = −0.39, *p* = 0.039). Interestingly anhedonia was correlated with greater self-reported depression in Alzheimer’s disease (DASS-D; *r* = −0.45, *p* = 0.02) but not in SD (DASS-D; *r* = −0.27, *p* = 0.20). Finally, anhedonia was significantly associated with carer burden exclusively in the SD group (ZBI: *r* = −0.60, *p* = 0.001; AD: *r* = 0.46, *p* = 0.20).

Fisher’s r to *Z* transformations were run to determine whether associations between anhedonia on the SHAPS and behavioural changes were stronger in the SD relative to the AD group. Overall, associations between anhedonia and functional impairment on the FRS (*Z* = 2.07, *p* = 0.02) and motivational changes on the CBI-R (*Z* = 2.28, *p* = 0.01) were significantly stronger in SD relative to AD patients.

### 3.6. Neuroimaging Results

#### 3.6.1. Group Differences in Grey Matter Intensity

**Figure 3:**
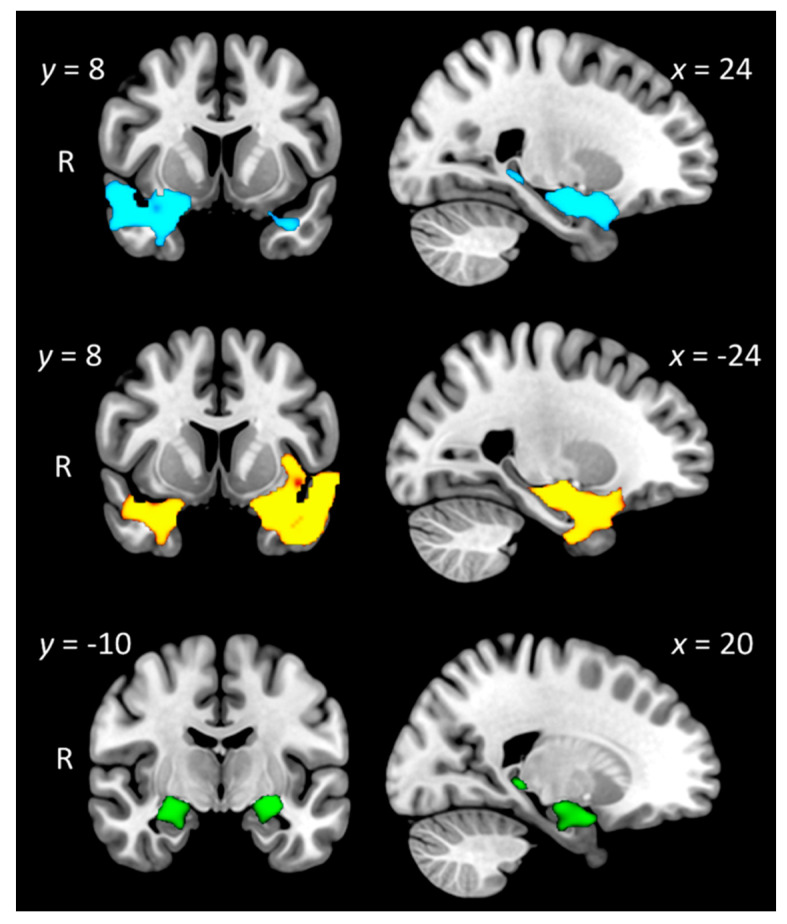
Regions of significant grey matter intensity decrease in right-predominant semantic dementia (SD-R: top panel; blue); left-predominant semantic dementia (SD-L: middle panel; yellow) and Alzheimer’s disease (AD: bottom panel; green) relative to Controls. Coloured voxels indicate regions that emerged as significant in the voxel-based morphometry analyses extracted voxelwise and corrected for family-wise error at *p* < 0.05. ACE-III Total, years in education, and scanning site included as nuisance variables in the analyses. Clusters are overlaid on the Montreal Neurological Institute (MNI) standard brain with x and y coordinates reported in MNI standard space. R = right. Figures created using MRIcroGL.

Figure 3 displays profiles of grey matter intensity decrease in each patient group relative to Controls (see Table 3 for full details). Briefly, SD-L patients displayed primarily left-sided temporal lobe atrophy, concentrated on the anterior temporal pole, including the anterior and posterior temporal fusiform cortex, amygdala, hippocampal, and parahippocampal regions. Significant grey matter intensity decrease was also observed in the right temporal pole and orbitofrontal cortices. Relative to Controls, SD-R patients displayed a largely right-predominant pattern of grey matter intensity decrease centred on the right ATL but spreading to include adjacent anterior temporal fusiform, anterior superior temporal, orbitofrontal, and insular cortices. Regions in the left hemisphere were also affected, including the left anterior temporal pole and amygdala, albeit to a lesser degree than on the right side. Direct comparisons between the SD groups failed to reveal any significant clusters at *p*_corrected_ = 0.05.

Finally, relative to Controls, AD patients displayed characteristic grey matter intensity decrease in bilateral medial temporal regions, including the bilateral hippocampus and amygdala, bilateral parahippocampal, and lateral temporal cortices, with additional involvement of the left orbitofrontal cortex, right lateral parietal regions, and the left thalamus.

These patterns of atrophy are consistent with independent reports in the literature for SD-L and SD-R variants and Alzheimer’s disease [61].

#### 3.6.2. Individual Differences in Magnitude and Lateralisation of Atrophy

**Figure 4:**
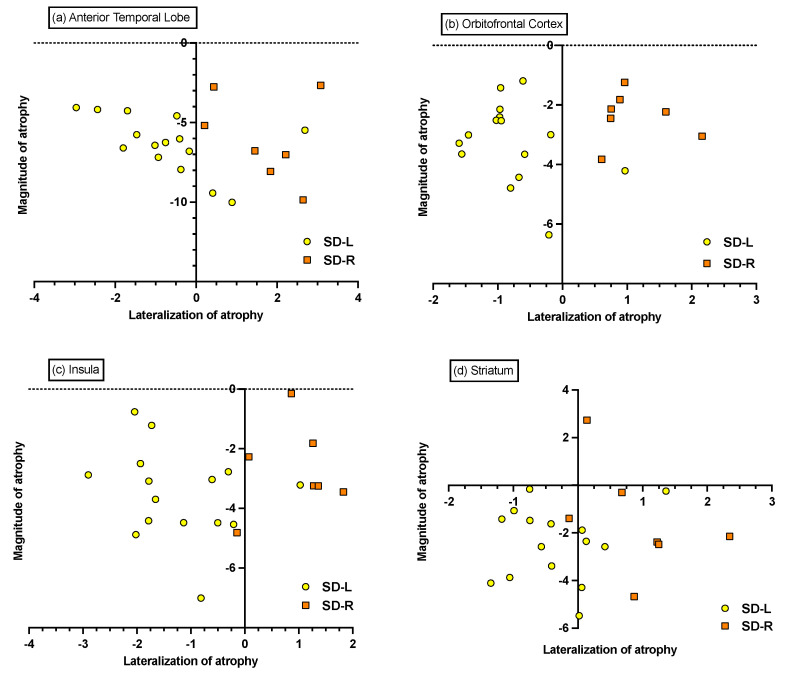
Magnitude and asymmetry of grey matter atrophy of (**a**) anterior temporal lobe, (**b**) orbitofrontal cortex, (**c**) insula, and (**d**) striatum in SD. All points indicate *z*-scored mean intensity values (*z*-scored relative to the Control group). The magnitude of atrophy index (i.e., sum of left and right intensity values) indicates the total amount of atrophy relative to Controls, with lower scores indicating greater total bilateral atrophy. The asymmetry of the atrophy index (i.e., difference of left and right intensity values) captures the lateralisation of atrophy, with positive scores suggesting right-lateralised atrophy, negative scores suggesting left-lateralised atrophy, and scores at/around zero indicating bilateral atrophy of relatively equal magnitude.

Visual inspection of the 4 ROIs (i.e., ATL, orbitofrontal cortex, insula, and striatum) revealed a relatively comparable magnitude of atrophy in both SD groups (Figure 4). The asymmetry indices suggest that a large number of SD-L patients display bilateral atrophy of comparable magnitude in the ATL and striatum. Similarly, while the majority of SD-R patients display predominantly right-lateralised atrophy across the ROIs, two SD-R cases exhibited near bilateral atrophy to the ATL, insula, and striatum.

#### 3.6.3. Associations between Grey Matter Intensity and Anhedonia on the SHAPS

**Figure 5:**
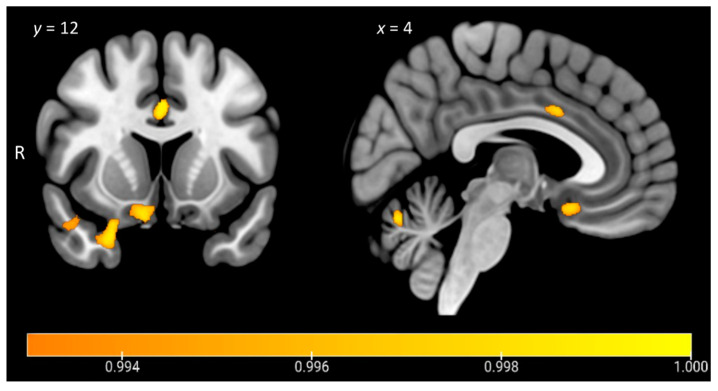
Voxel-based morphometry analyses showing regions of significant grey matter intensity that correlate with anhedonia severity in all patients combined (*n* = 45). Coloured voxels indicate regions that emerged as significant in the analyses, extracted voxelwise, and corrected for a false discovery rate at *q* < 0.05. ACE-III Total, years in education, and scanning site included as nuisance variables in the analyses. Clusters are overlaid on the Montreal Neurological Institute (MNI) standard brain with x and y coordinates reported in MNI standard space. R = right. Figures created using MRIcroGL. Colour bar represents grey matter intensity.

Figure 5 and Table 4 display the results from the VBM correlation analyses exploring associations between carer ratings of current anhedonia on the SHAPS across the entire patient cohort (*n* = 45). Lower scores on the SHAPS, reflecting greater levels of anhedonia, were associated with grey matter intensity decrease in a relatively restricted set of regions centred on right orbitofrontal and temporopolar cortices, bilateral posterior temporal cortices, as well as the anterior cingulate gyrus, parahippocampal gyrus, and cerebellum, bilaterally.

#### 3.6.4. A Unique Role for Right Temporal Regions in Anhedonia in SD

A forward stepwise multiple regression analysis was run to explore whether grey matter intensity across the four ROIs (i.e., ATL, orbitofrontal cortex, insula, and striatum) in the left and right hemisphere (i.e., 8 ROIs in total) predicted current anhedonia scores in the combined SD group (*n* = 22). The resultant model indicated that grey matter intensity of the right ATL significantly predicted anhedonia severity in SD (R-squared = 0.21; *F*(1,71) = 18.67, *p* < 0.001; VIF = 1).

#### 3.6.5. Mediation Analysis

Finally, a mediation analysis was conducted to clarify whether left ATL grey matter intensity mediated the effect of right ATL contributions to anhedonia scores in the combined SD group. The indirect effect was tested using a percentile bootstrap estimation approach with 5000 samples [62] implemented with PROCESS macro v.3 in SPSS [63]. The addition of the left ATL into the model did not sufficiently explain anhedonia performance, *t* = 0.21; *p* = 0.83, 95% CI (−20.6–25.5), nor did it improve model fit as the R-square value only marginally increased from 0.2082 (right ATL model) to 0.2087 (left and right ATL model). Importantly, only ~6% of the total effect of the model was mediated by left ATL atrophy, and the indirect coefficient (i.e., left ATL mediation) was not statistically significant (B = 2.33, SE = 12.29, 95%CI (−22.37, 25.87), partially standardised β = 0.34). These findings suggest that right temporal lobe structures make a unique contribution to the emergence of anhedonia in SD.

## 4. Discussion

The objective of this study was to explore how degeneration of right hemispheric brain structures impacts the hedonic experience in SD, focusing on the less common right-predominant presentation (SD-R). Using the SHAPS as a well-established assay of hedonic tone, we found clinically significant anhedonia exclusively in the SD-R group relative to Controls and patients with Alzheimer’s disease (AD). Whole-brain voxel-based morphometry analyses revealed that loss of hedonic tone across the entire patient cohort was associated with grey matter intensity decrease in a predominantly right-lateralised frontotemporal brain network. Key regions included the right orbitofrontal cortex and right temporal pole, along with the anterior cingulate cortex, and the cerebellum, bilaterally. While many of these regions have been reliably implicated in the neuropsychiatric literature on anhedonia, in particular the orbitofrontal and anterior cingulate cortices [64], it is notable that we did not find any striatal involvement in the current study.

Our regression and mediation analyses instead demonstrated a unique role for the right ATL in the origin of anhedonia in SD, resonating with a number of studies implicating degeneration of the right ATL in the genesis of socioemotional disturbances in this syndrome [36]. Interestingly, while SD-R were rated as displaying higher levels of anhedonia relative to the AD group, no significant differences were observed between SD-R and their SD-L counterparts. Inspection of the SD-L group distributions, however, revealed considerable variability in anhedonia severity, suggesting marked heterogeneity in this group. We suggest that the SD-L sample likely comprises distinct subgroups that differ in their capacity for hedonic experience depending on disease staging. This proposal was borne out in a post hoc correlation analysis whereby anhedonia severity in the SD-L group was significantly associated with disease duration (*r* = −0.48, *p* = 0.04) and most likely reflects the progression of atrophy from the left into the contralateral temporal lobe. While there is a tendency to dichotomise SD into left- or right-sided variants, this binary classification obscures the fact that, unless assessed very early in the disease trajectory, bilateral atrophy is invariably present in these patients [65,66]. Our findings indicate that patients traditionally characterised as having a language disorder can present with marked neuropsychiatric changes [67] but that the severity of these changes likely differs depending on disease staging. Interestingly, patients with predominantly right-temporal atrophy displayed elevated self-rated depression relative to Controls, suggesting a link between right temporal dysfunction and neuropsychiatric and mood disturbances more broadly [68], although this proposal requires formal testing. Our findings bear relevance to early studies reporting apparent emotional indifference in lesion cases with right-sided, but not those with left-sided brain injury [69] and underscore the possible privileged role of the right hemisphere in the processing of emotional responses [70].

Considering next the role of prefrontal regions, we found significant involvement of the right orbitofrontal cortex and bilateral anterior cingulate cortex in relation to anhedonia. A large body of evidence suggests an important role for the orbitofrontal cortex in the computation and processing of expected reward value and in modulating emotional responses upon the receipt of an expected reward [64]. Moreover, orbitofrontal cortex dysfunction is reliably implicated in the emergence of negative symptoms such as flattening of affect in neuropsychiatric disorders [71] as well as emotional components of apathy in frontotemporal dementia [72]. The spread of atrophy into right orbitofrontal regions in SD may disrupt the affective tagging of stimuli as rewarding [73,74] or block the endogenously driven anticipation of future events and goal states [75]. Previous studies have demonstrated a profound inability to envisage future events in SD; however, these difficulties have been linked to ATL rather than orbitofrontal degeneration [76,77]. It remains unclear how the impaired capacity to imagine oneself in the future relates to anticipatory aspects of anhedonia; however, we suggest this will be an important area for future studies. Given the importance of pleasure and motivation for goal-directed behaviour, the coalescence of apathy, impaired future thinking, and anhedonia in SD warrants serious consideration in light of recent reports of depression and suicidal behaviour as a reaction to future thinking deficits in this syndrome [78].

We further found significant associations between the anterior cingulate cortex and anhedonia severity. The anterior cingulate cortex has previously been implicated in emotional processing [79] and reward-based decision making [80], while decreased activation of this region has been observed during reward processing in patients with a major depressive disorder [81]. Cortical thinning of the anterior cingulate cortex has further been implicated in the decline of emotional processing and social behaviour in SD patients [33]. Importantly, this region forms a key node of the brain’s salience network, a large-scale network that plays a crucial role in identifying the most biologically and cognitively relevant endogenous and external stimuli to guide adaptive behaviour [82,83]. A consistent observation in the literature is that of pronounced right-hemisphere dominance for salience processing, centred on the right anterior insula, anterior cingulate cortex, and medial prefrontal regions [84]. The right anterior insula has been put forward as a key integrative hub of the salience network due to its anatomical location and bilateral connections with prefrontal and striatal brain regions critically involved in reward processing and motivation [85]. While dysfunction of the salience network is not traditionally associated with SD, our group-based atrophy analysis revealed significant degeneration of core nodes of this network exclusively in the SD-R group, including the right orbitofrontal cortex, right anterior insular cortex, and right ventral striatal regions. Our brain-behaviour covariate analyses, however, did not reveal significant associations between the right insular cortex and anhedonia; however, this may reflect a lack of power due to the rarity of the SD-R syndrome. Future longitudinal studies exploring how the progressive degeneration of right salience network regions impacts reward-processing disturbances in SD will be critical to determine the temporal course and neurobiological mechanisms of these symptoms. In this regard, we suggest that incorporating measures of structural connectivity will further enable us to consider how dysregulation of extended brain networks [86] impacts the capacity for hedonic tone in this syndrome [87].

A number of methodological issues and future directions warrant consideration. Firstly, the current sample of SD patients was composed of decidedly more SD-L relative to SD-R patients, reflecting the rarity of the SD-R syndrome. Future investigations with larger samples will be important to confirm our findings and explore between-group differences further. Secondly, while the SHAPS remains one of the most well-established tools to query anhedonia, it fails to capture the multifaceted nature of the hedonic experience [88]. Mounting evidence indicates that anhedonia is a multidimensional construct and likely reflects the breakdown of a number of distinct yet interacting components, including anticipation, motivation, and consumption [64]. As such, it will be important to distinguish between different components of anhedonia (e.g., anticipatory versus consummatory) and to determine how anhedonia relates to reward-related motivational disturbances in SD. For example, given the significant prefrontal contributions uncovered here, we might predict that the loss of pleasure in SD primarily reflects disruption of an anticipatory, rather than consummatory, mechanism [64]. Adopting a multidimensional approach will enable us to parse anhedonia into functionally relevant subcomponents and to determine the role of temporal, prefrontal, and striatal pathways in anticipating and responding to reward receipt versus consummatory stages of hedonic processing [64]. From a clinical perspective, recognising anhedonia as a potential indicator of right temporal lobe atrophy may provide a marker of disease staging in SD, enabling us to better differentiate between left- and right-sided presentations and to anticipate the emergence of neuropsychiatric symptoms with disease progression [89]. Longitudinal studies will be particularly important to determine at what stage of the SD-L disease trajectory anhedonia manifests and to ensure that carers are equipped to anticipate and respond to these symptoms. A final limitation of our study is that it was not possible to ascertain the relationship between anhedonia and left-right asymmetry in SD at initial symptom onset as our sample was recruited as part of an ongoing longitudinal study. As such, the anhedonia profiles presented here may not necessarily be representative of the earliest stages of the SD disease process. Future work exploring anhedonia in SD at its mildest form would serve to clarify the respective contributions of the left versus right hemispheres to anhedonia, although recruiting such cases is challenging.

## 5. Conclusions

In conclusion, this study demonstrates an important role for right hemispheric structures in modulating anhedonia severity in SD. Our findings offer important insights into potential hemispheric lateralisation of motivational disturbances in dementia, paving the way for future studies to explore this long-neglected clinical symptom.

## Figures and Tables

**Table 1:** Demographic, clinical, and cognitive characteristics of the study cohort.

	SD-R (*n* = 8)	SD-L (*n* = 20)	AD (*n* = 30)	Control (*n* = 30)	Group Effect	Post Hoc Comparisons
Age, years	64.9 (8.1)	67.8 (7.4)	66.2 (8.2)	64.8 (7.1)	F = 0.64 ^n/s^	
Education, years	12.6 (3.3)	13.5 (3.2)	12.8 (2.8)	14.6 (3.4)	F = 1.78 ^n/s^	
Sex, M:F	3:5	9:11	16:14	15:15	χ = 0.79 ^n/s^	
Disease duration, years	5.7 (1.8)	4.6 (2.3)	4.8 (1.7)	--	F = 0.86 ^n/s^	
ACE-III Total (100)	66.9 (17.7)	67.2 (12.5)	71.7 (7.8)	95.3 (3.3)	F = 50.5 ***	Controls > SD-L, SD-R, AD
FRS Rasch ^a^	−0.1 (1.1)	1.2 (1.7)	0.6 (1.3)	--	F = 2.64 ^n/s^	
CBI-R Total (%)	35.6 (15.2)	22.2 (12.5)	27.4 (15.1)	--	F = 2.36 ^n/s^	
CBI-Motivation	46.4 (31.7)	32.9 (26.6)	35.0 (27.3)	--	F = 0.63 ^n/s^	
DASS-Depression (21)	6.3 (5.8)	3.9 (3.3)	3.7 (3.4)	1.5 (1.8)	F = 5.57 **	SD-R > Controls

*Note*. Values are in the format mean (standard deviation) unless otherwise specified. Maximum score for each test shown in parentheses where appropriate. ^a^ Lower scores denote greater levels of functional impairment on FRS. ** *p* < 0.005; *** *p* < 0.0001; n/s = not significant; -- = not applicable; ACE-III = Addenbrooke’s Cognitive Examination third edition; AD = Alzheimer’s disease; CBI-R = Cambridge Behavioural Inventory—Revised; DASS = Depression, Anxiety, and Stress Scale; F = Female; FRS = Frontotemporal Dementia Functional Rating Scale; M = Male; SD-L = left-predominant semantic dementia; SD-R = right-predominant semantic dementia. FRS not available for 1 SD-R, 4 SD-L and 1 Alzheimer’s disease; CBI-R not available for 1 SD-R, 1 SD-L and 1 Alzheimer’s disease; DASS not available for 2 SD-R, 2 SD-L and 4 Alzheimer’s disease.

**Table 2:** Pearson’s *r* correlations exploring associations between carer-rated anhedonia severity on the SHAPS and clinical and behavioural measures in the patient groups.

	SD Combined (*n* = 28)	Alzheimer’s Disease (*n* = 30)
Disease duration	−0.32	−0.15
FRS Rasch Score ^a^	0.79 ***	0.46 *
ACE-III Total	0.33	0.11
DASS-Depression	−0.27	−0.45 *
CBI-R Motivation	−0.78 ***	−0.39 *
CBI-R Total	−0.70 ***	−0.51 **
ZBI	−0.60 **	−0.25

*Note*. ^a^ Lower scores denote greater levels of functional impairment on FRS. * *p* < 0.05; ** *p* < 0.005; *** *p* < 0.0001; ACE-III = Addenbrooke’s Cognitive Examination third edition; CBI-R = Cambridge Behavioural Inventory—Revised; DASS = Depression, Anxiety, and Stress Scale; FRS = Frontotemporal Dementia Functional Rating Scale; SD = Semantic Dementia; ZBI = Zarit Burden Interview. FRS not available for 5 SD and 1 Alzheimer’s disease; CBI-R not available for 2 SD and 1 Alzheimer’s disease; DASS not available for 4 SD and 4 Alzheimer’s disease; ZBI not available for 3 SD and 2 Alzheimer’s disease.

**Table 3:** Voxel-based morphometry results showing regions of significant grey matter intensity decrease in Alzheimer’s disease, left-predominant semantic dementia, and right-predominant semantic dementia, relative to Controls.

Contrast	Regions	Side	Cluster Size	Peak MNI Coordinates			*t* Value
				x	y	z	
Controls—SD-R	Temporal pole, orbitofrontal cortex, anterior temporal fusiform cortex, anterior superior/middle temporal gyrus, insular cortex, amygdala, putamen, pallidum, hippocampus, parahippocampal gyrus	R	2954	34	14	−36	3.97
	Temporal pole	L	136	−36	8	−30	3.01
	Hippocampus, amygdala	L	60	−28	−6	−24	3.46
	Hippocampus, thalamus	R	58	26	−34	−6	2.59
Controls—SD-L	Anterior and posterior temporal fusiform cortex, anterior and posterior parahippocampal gyrus, hippocampus, amygdala, temporal occipital fusiform cortex, temporal pole, orbitofrontal cortex, insular cortex, putamen, superior/middle temporal gyrus	L	5615	−30	−8	−44	3.87
	Temporal pole, orbitofrontal cortex, anterior parahippocampal gyrus, anterior and posterior temporal fusiform cortex, amygdala, hippocampus	R	1793	38	16	−36	3.87
Controls—AD	Anterior parahippocampal gyrus, temporal pole, amygdala, hippocampus	R	387	24	2	−20	3.58
	Amygdala, hippocampus, temporal pole, anterior parahippocampal gyrus, orbitofrontal cortex, putamen, pallidum	L	347	−26	−12	−14	3.58
	Angular gyrus, superior parietal lobule, supramarginal gyrus	R	67	23	37	55	3.35
	Thalamus, hippocampus	R	65	18	−34	0	3.58
SD-L—SD-R	No significant clusters						
SD-R—SD-L	No significant clusters						

*Note*. Age, total education, and scanning site included as nuisance variables in all contrasts. Clusters were extracted voxelwise at *p* < 0.05 corrected for family-wise error. MNI = Montreal Neurological Institute; L = left; R = right; B = bilateral; AD = Alzheimer’s disease; SD-L = semantic dementia left variant; SD-R = semantic dementia right variant.

**Table 4:** Voxel-based morphometry results showing regions of significant grey matter intensity decrease that covary with anhedonia severity across the entire patient cohort (*n* = 45).

Contrast	Regions	Side	Cluster Size	Peak MNI Coordinates			*t* Value
				x	y	z	
SHAPS	Orbitofrontal cortex, temporal pole, subcallosal cortex	R	351	18	32	−22	3.62
	Temporal pole, orbitofrontal cortex	R	272	24	10	−28	3.48
	Temporal occipital fusiform cortex, posterior temporal fusiform cortex, posterior parahippocampal gyrus, lingual gyrus	R	269	34	−46	−12	3.48
	Cerebellum	B	199	−6	−68	−22	3.86
	Posterior temporal fusiform cortex, posterior parahippocampal gyrus	L	134	−36	−42	−10	3.18
	Anterior cingulate gyrus, left paracingulate gyrus	B	102	0	12	34	3.86

*Note*. Total ACE-III score, education in years, and scanning site included as nuisance variables in all contrasts. Clusters were extracted voxelwise, corrected for a false discovery rate at *p* < 0.05 with a cluster threshold of 50 contiguous voxels. SHAPS = Snaith–Hamilton Pleasure Scale; ACE-III = Addenbrooke’s Cognitive Examination third edition; MNI = Montreal Neurological Institute; L = left; R = right; B = bilateral.

## Data Availability

The ethical requirement to ensure patient confidentiality precludes public archiving of our data. Researchers who would like to access the raw data should contact the corresponding author who will liaise with the ethics committee that approved the study, and accordingly, as much data that is required to reproduce the results will be released to the individual researcher. No part of the study procedures or analyses were preregistered prior to the research being undertaken. The code used for this project has been made available for review on the Open Science Framework website (https://osf.io/jzmnr/ (accessed on 27 July 2021)).
